# The Role of miRNAs as Key Regulators in the Neoplastic Microenvironment

**DOI:** 10.4061/2011/839872

**Published:** 2011-04-06

**Authors:** K. K. Wentz-Hunter, J. A. Potashkin

**Affiliations:** ^1^Biological, Chemical and Physical Sciences Department, College of Arts and Sciences, Roosevelt University, Chicago, IL 60605, USA; ^2^Department of Cellular and Molecular Pharmacology, The Chicago Medical School, Rosalind Franklin University of Medicine and Science, 3333 Green Bay Road, North Chicago, IL 60064, USA

## Abstract

The neoplastic microenvironment has been recognized to play a critical role in the development of cancer. Although a large body of evidence has established the importance of the cancer microenvironment, the manners of crosstalk between it and the cancer cells still remains unclear. Emerging mechanisms of communication include microRNAs (miRNAs). miRNAs are small noncoding RNA molecules that are involved in the posttranscriptional regulation of mRNA. Both intracellular and circulating miRNAs are differentially expressed in cancer and some of these alterations have been correlated with clinical patient outcomes. The role of miRNAs in the tumor microenvironment has only recently become a focus of research, however. In this paper, we discuss the influence of miRNAs on the tumor microenvironment as it relates to cancer progression. We conclude that miRNAs are a critical component in understanding invasion and metastasis of cancer cells.

## 1. Introduction

The belief that cancer cells are the sole dictators of their proliferative, invasive and migratory abilities has been revised. In its place is the view that the six hallmarks of cancer cells, namely self-sufficiency in growth signals, insensitivity to antigrowth signals and self-renewal, evasion of apoptosis, limitless replication potential, angiogenesis, invasion and metastasis, result from heterotypic signaling of cancer cells with its microenvironment [1]. Carcinoma progression and prognosis are dependent on the interaction of cancer epithelial cells with, and the recruitment of, tumor stroma, immune cells, and vascular networks as well as the alteration of the extracellular matrix [ECM] components (reviewed in [2–4]).

How do cancer cells influence normal cells to abandon their homeostatic activities and instead support the neoplastic nature of the tumor? Obviously, there must be some method of communication between the cancer cells and their microenvironment. The dynamic crosstalk between cancer cells and normal cells in the microenvironment is a crucial point in the progression of disease. One manner of cell-cell communication is through the secretion of molecules and paracrine signaling. Molecules of secretion are no longer limited to cytokines, chemokines, growth factors, and other protein molecules but now include miRNA species. MiRNAs are pleiotropic regulators of gene expression that regulate various normal cellular processes as well as play a role in disease progression, especially cancer. Some intracellular and circulating miRNA species are dysregulated in cancer, leading to altered expression that in some cases has been correlated with clinical outcomes for patients. Furthermore, a number of recent studies have investigated the use of miRNAs as noninvasive biomarkers for cancer diagnosis and prognosis. However, the understanding of the cellular consequences in the microenvironment of these differentially expressed miRNAs is just beginning to be elucidated. In this paper, we will specifically focus on carcinomas and miRNA regulation of the associated microenvironments including the tumor stroma, ECM components, and hypoxia.

## 2. miRNAs and Cancer-Associated Fibroblasts

The tumor stroma is composed of a number of different cell types including fibroblasts, myofibroblasts, adipocytes, endothelial cells, pericytes, and immune cells. The tumor stroma is essential not only in the survival of neoplasms but the ability of these tumors to evolve invasiveness and metastatic ability. Early studies appeared to indicate that cancer-associated fibroblasts (CAF) also accumulated genetic mutations much like neoplastic cells [5–8]. However, further research has revealed that mutations in CAFs are rare events indicating that other manners of altering gene expression profiles in these cells must exist [9, 10]. A possible emerging mechanism for the control of gene expression within the tumor stroma by the cancer epithelium is the differential expression and transfer of miRNA species (summarized in Table 1). 

A recent bladder cancer study has identified specific miRNA profiles in CAFs [11]. Five independent fibroblast cell lines were established from invasive bladder cancer and compared to normal bladder and foreskin fibroblasts in a miRNA microarray and validated in quantitative polymerase chain reaction (qPCR) assays. These studies observed an increase in miR-16 and miR-320 in CAFs in comparison to normal bladder fibroblasts. Surprisingly, miRNAs such as miR-16 and miR-320 have been indicated to function in a tumor suppressor manner and shown to be downregulated in certain cancers. The low expression of miR-16 and miR-320 in colorectal and pancreatic cancers [12, 13] is believed to be involved in cell proliferation [14]. The identification of these upregulated miRNAs in CAFs may indicate an initial compensatory response by the CAFs in an attempt to block cellular proliferation. Alternatively, it is possible that the upregulated miRNAs in CAFs facilitate tumor survival or progression. Additional investigations are necessary to determine the potential consequences of miRNAs upregulated in one cell population and downregulated in another within a tumor.

In contrast to the upregulated miRNAs, miR-143 and miR-145 are downregulated in CAFs compared to foreskin fibroblasts. There was no significant decrease in miR-143 and miR-145 between CAFs and normal bladder fibroblasts, however, although a trend was reported. MiR-143 and miR-145 are located on chromosome 5 approximately 1.3 kb apart and are most likely transcribed as a cluster; therefore, it is not surprising that similar trends are observed. Previous studies in bladder cancer have shown decreased levels of miR-145 correlated with a decrease in apoptotic ability suggesting that a decrease in miR-145 in CAFs may also contribute to evasion of apoptosis in the microenvironment [15–17]. A similar correlation with miR-143 has not been reported.

Another study looking at ten different CAF cell lines from endometrial cancers identified eleven differentially expressed miRNAs in the CAFs in comparison to normal fibroblasts [18]. Of these eleven miRNAs identified in microarrays, five were validated as statistically significant in qPCR. These included increased expression of miR-503 and miR-424, which are present in a cluster of miRNAs found on chromosomal location Xq26.3. Other upregulated miRNAs were miR-29b and miR-146a. One miRNA, miR-31, was shown to be downregulated in 9 out of 10 CAF cell lines. In addition to qPCR validation of miRNA species, these five miRNAs were shown to have significant negative correlation with their targets genes in microarray analysis of total RNA from CAFs. For miR-31, 39 targets were identified as predicted upregulated proteins in these cell lines, while for the miR-503 and miR-424 cluster, 139 combined protein targets were predicted to be downregulated. This correlation provides additional evidence suggesting a functionally important role of these miRNAs in CAF gene regulation.

**Figure 1:**
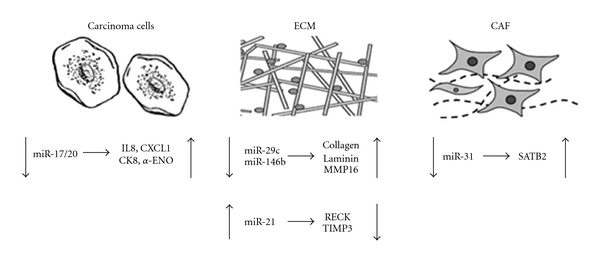
Validated miRNAs and their gene targets in the microenvironment. In carcinoma cells, the loss of miR-17/20 is involved in heterotypic signaling through the upregulation of cytokines IL-8 and CXCL-1 as well as plasminogen activators CK8 and *α*-ENO. In the ECM, decreased amounts of miR-29c are found to result in an increase in collagens and laminin leading to alteration in ECM composition conducive to invasion and migration. In addition, miR-146b is also decreased resulting in increased activity of MMP16 in ECM degradation. In contrast, miR-21 levels are elevated resulting in inhibition of RECK and TIMP3, important metalloproteinase inhibitors. In the CAF, although multiple miRNAs are differentially expressed, to date only a decrease in miR-31 has been validated to be directly involved in an upregulation of SATB2 resulting in increased invasiveness and migration.

Further analysis of miR-31 demonstrated that a number of the predicted target genes for miR-31 are involved in cellular movement. Proteins related to transcriptional control found to be overexpressed included CCNJ, ELAVL1, and ENY2. Genes involved in cellular movement and transport correlated to lower levels of miR-31; they included RHOBTB1 and CLASP2 involved in cytoskeletal organization, VAMP4 in endosomal transport and STX12 in phagocytosis. A gene functioning in cellular transformation and anchorage-independence, TACC2, is also a putative target of miR-31. SATB2 demonstrated the greatest fold induction of protein in these cells and is known to function in chromatin remodeling and transcription regulation. Direct evidence of SATB2 as a target for miR-31 was provided through luciferase reporter assays (Figure 1). When conditioned media from fibroblasts overexpressing miR-31 was incubated with endometrial carcinoma cells, their migration and invasion potential was decreased although there was no effect on cell proliferation. The opposite was observed when SATB2 was overexpressed in these cells indicative of a direct role of miR-31 dysregulation in tumor stromal cells and the progression of carcinomas. Taken together, these induced genes can provide CAFs with some of the hallmarks of cancer cells such as anchorage-independence and enhanced cellular movement and as such potentially serve as a means by which CAFs act as support cells to the carcinoma. 

However, there is some conflicting evidence on the role of miR-31 in carcinomas. Upregulation of this miRNA was found in colon cancer [19–22] and squamous cell carcinomas of the tongue [23]. Interestingly, in colon cancer studies not only was miR-31 upregulated but this upregulation was found to be associated with poorer clinical outcomes and increased invasiveness [22]. Although this seems like a potential contradiction for the role of miR-31 in tumor progression, an important variable between this study and that of Aprilikova and colleagues is that the colon cancer study evaluated the carcinoma cells and not the supporting tumor stroma. Then again, there is some support for downregulation of miR-31 in breast cancer [24, 25], gastric cancer [26], and urothelial cancers [27]. In addition to the differences in the type of cancer under investigation in these studies, there are differences in methods used to retrieve and prepare the tissues. Other details of the methods used in the various studies could also have profound effects on the data including the choice of normalizer used for quantification of the qPCR assays [28, 29]. Such contradictory reports of miR-31 expression in cancer cells and the microenvironment further highlight the importance of not only identifying differential miRNA expression but the elucidation of validated target genes. 

The previously cited investigations identified miRNAs that are dysregulated in CAFs and may contribute to tumor progression. These studies did not look at the effects of miRNA signals from cancer epithelial cells on the tumor microenvironment. A recent study by Yu and colleagues has done just that [30]. Through their evaluation of three highly invasive cell lines and four non-invasive cell lines from breast cancers, they have identified a miRNA cluster, miR-17/20, involved in heterotypic signaling. When miR-17/20 is expressed in cancer cells, their ability for migration and invasion is suppressed. In fact, when conditioned media from miR-17/20 expressing cells was incubated with the highly invasive cell line, MDA-MB-231, the ability of these cells to migrate and invade across wounds or 3D collagen gels was greatly inhibited. Further analysis of the role of miR-17/20 in breast cancer cells determined that this miRNA cluster was involved in the regulation of cellular secretion that altered the cellular microenvironment. When miR-17/20 levels are decreased, cancer cells increase secretion of cytokines IL-8 and CXCL-1 and proteins CK8 and alpha-ENO (Figure 1). While secreted IL-8 and CXCL-1 have direct effects on neighboring cells through interaction with their respective receptors, CK8 and alpha-ENO effects are indirect as they both act on plasminogen activation [31, 32]. The increase in plasmin from plasminogen activation is an important player in the process of ECM degradation that ultimately facilitates migration and invasion. Moreover, an increase in plasminogen activation has clinical importance in breast cancer patients as it correlates with increased invasiveness and poorer prognosis [33]. This is the first line of evidence to show that miRNAs are important in heterotypic signaling in the microenvironment of the tumor and to correlate such a role to clinical outcome.

This information taken together highlights the importance of determining where miRNA alterations originate in tumor cells or the tumor stroma and establishing what factors are responsible for these altered miRNA levels. Other questions that deserve experimental attention are to evaluate both primary and metastatic tumors to determine if miRNA dysregulation is sustained or only associated with tumor progression. In other words, are miRNA alterations that we are currently detecting simply stills in an ever changing landscape of tumor progression?

## 3. ECM Composition Influenced by miRNAs

The ECM is a key regulator in neoplasmic cell growth and mobility. Alteration of the ECM to allow cell invasion, migration, and angiogenesis is essential for a cancer's ability to move from a localized, primary site. It is well established that signals such as increased matrix metalloproteinases (MMPs) or decreased tissue inhibitors of metalloproteinases (TIMPs) released into the tumor microenvironment play a critical role in the reorganization of the ECM and thus contribute to cancer metastatic ability. But what roles do miRNAs play in MMP and TIMP expression? 

MMPs function to degrade ECM proteins and process bioactive molecules. In cancer progression, these functions are important not only in the release of signaling molecules from the ECM but in providing an avenue for migration, invasion, and angiogenesis. MiR-21 has been shown to be a major player in such migration and invasion in multiple cancers including gliomas, cholangiocarcinomas, and breast cancers [24, 34–37]. In gliomas, miR-21 levels have been correlated with higher-grade tumors including glioblastomas [38]. Targets for miR-21 in these cells have identified two MMPS inhibitors, RECK and TIMP3, that inversely correlate with the miRNA (Figure 1). RECK is a membrane-anchorage regulator while TIMP3 is an ECM-bound protease inhibitor. When these proteins are downregulated in the presence of miR-21, there is a subsequent increase in MMP activity and invasiveness of the glioma. In addition, TIMP3 has been previously shown to be proapoptotic so the loss of this inhibitor also allows for the evasion of apoptosis. Supporting evidence for the ability of miR-21 to act as a direct inhibitor of TIMP3 function was substantiated by work by Selaru and colleagues in human cholangiocarcinomas [36]. In contrast, although their work has demonstrated that miR-21 was upregulated in these tumors, it was not correlated with location or grade of tumor. Nevertheless, there were significant increases in miR-21 expression and corresponding loss in TIMP3 message in all tumor cell lines evaluated although a direct role in invasiveness was not assessed in their study.

Although the previous studies did show a relationship between miR-21 expression and TIMP3 levels, none showed that miR-21 was directly inhibiting TIMP3 expression through binding to predicted sites in the 3′UTR of the transcript. Such conclusive evidence has recently been presented in breast cancer cells [37]. Luciferase reporter assays using the TIMP3 3′UTR and miR-21 showed a significant increase in activity when breast cancer cell line MDA-MB-231 was transfected with both species. In addition, their work further confirmed the association between miR-21 increases, loss of TIMP3, and cell invasion both in culture and in lymph node positive tissue specimens. Taken together, these studies provide strong evidence for the role of miR-21 expression in altering the ECM microenvironment of cancer cells allowing invasion and metastasis.

Not surprisingly, other miRNAs are also currently being identified as associated with ECM reorganization in relationship to cancer. Much like miR-21, miR-146b has also been demonstrated to play a role in glioma cell invasion [39]. However, miR-146b levels are found to be lower in glioblastoma cell lines. Thus, miR-146b does not work through the suppression of ECM inhibitors but its loss allows the upregulation of MMPs. Direct association of miR-146b with the 3′UTR of MMP16 was shown to be linked with a loss of MMP expression (Figure 1). When miR-146b levels are low, migration and invasion are increased in several glioma cell lines. 

Another downregulated miRNA, miR-29c, was also shown to alter the level of ECM protein in a highly invasive cancer, nasopharyngeal carcinoma [40]. Experiments using 31 tumor specimens identified miR-29c to have an approximate 5-fold decrease in relation to normal specimens. Evaluation of potential targets for miR-29c revealed 10 ECM component genes to be upregulated in these same tumors. These genes included collagen types 3A1, 4A1, and 5A1 as well as laminin and thymine-DNA glycosylase. Direct targeting of these genes was confirmed through luciferase activity. The ECM components mentioned above have been implicated in cell migration and invasion by assisting in matrix renewal allowing for tumor mobility. In addition, increases in deposits of collagen and laminin have clinical association with metastases in a variety of solid tumors [41].

The above data illuminate the extensive role that miRNAs are predicted to have on ECM composition and remodeling during tumor progression. As evident, there are many different avenues including inhibition of TIMPs, activation of MMPs, and alteration of ECM component secretion for miRNAs to promote changes in the ECM that would be beneficial to the tumor. Primary tumors that have acquired altered miRNA levels will utilize these changes to further their destructive migratory and invasive properties.

## 4. The Role of miRNAs in the Hypoxic Response

The survival of a tumor is dependent on an hypoxic microenvironment, which increases its invasiveness and resistance to drug treatment [34]. A signature of hypoxia-inducible miRNAs has been identified which includes miR-21, 23a, 23b, 24, 26a, 26b, 27a, 30b, 93, 103, 103, 106a, 107, 125b, 181a, 181b, 181c, 192, 195, 210, and 213 [42]. Results from other studies also found that miR-210, 30b, 93, and 181b were induced under hypoxic conditions [43–45]. In addition, miR-429, 498, 572, 563, 637, 628 were found to be induced during hypoxia using a different type of microarray [44]. Some miRNAs are downregulated under hypoxic conditions including miR-122a, 565, 195, 30e-5p, 374, 19a, 101, 424, 29b, 186, 141, 320, 422b, and 197 in squamous cell carcinoma cells, miR-15b, 16, 20a, 20b, 30b, and 224 in carcinoma of nasopharyngeal epithelial cells, and miR-424 in trophoblasts [43–45]. There is also evidence that the response to hypoxia may be cell-specific. For example, let-7e, 7g and 7i are hypoxia-inducible in squamous cell carcinoma [44], whereas let-7a, 7c, 7d, 7e, 7f, and 7g are downregulated by hypoxia in nasopharyngeal carcinomas [43]. Further proof of cell specificity in the response to hypoxia is provided by results that showed let-7f, 7g, and 7i have contrasting expression levels in different colon and breast cancer cell lines [46]. 

Most of the hypoxia inducible miRNAs are also overexpressed in solid tumors suggesting that the induction of miRNAs in hypoxia initiates a signaling pathway that leads to tumor survival and/or proliferation [42]. A good example is glioblastomas, which have a hypoxic microenvironment [47, 48]. As previously mentioned, miR-21 is overexpressed in glioblastoma [38, 49]. In addition to its role in ECM modifications through the inhibition of TIMP3, this overexpression also plays a role in apoptosis evasion in glioblastoma cells, suggesting a role in cell survival. Therefore it is likely that the hypoxic microenvironment of glioblastomas lead to overexpression of miR-21, which modulates expression of transcripts that are needed for the tumors survival. In another example, colon cancer miR-107 can inhibit p53 regulation of hypoxic signaling and tumor angiogenesis [50].

Another area that needs further investigation in order to understand how miRNAs are regulating the hypoxic response and its role in cancer is identification of the targets of these regulators. Bioinformatic analysis of predicted targets suggest that the hypoxic regulated miRNAs may play a role in regulating apoptosis, cell proliferation, vascularization, and the response of cancer to chemotherapy. Unfortunately only a few of the predicted targets have been experimentally tested (see below). One example that has been tested is HMGA2 (high-mobility group A2), which has been shown to play a role in how the tumor responds to chemotherapy. Expression of HMGA2 is reduced in a hypoxic environment and it is likely that miR-98, let-7g, 7e, and 7i are at least partially responsible for this regulation [45]. More recently, it was shown that vascular endothelial growth factor (VEGF) expression is mediated by hypoxia-inducible factor HIF-1 and STAT3 in a miR-20b-dependent manner in MCF-7 breast cancer cells under conditions that mimic hypoxia [51]. The role of miR-20b in modulating VEGF expression in an oxygen dependent manner was confirmed in a separate study [52].

Several studies suggest that miR-210 is key regulator of the HIF response to hypoxia in cancer (reviewed in [53]). In one study, the expression of miR-210 and miR-373 were induced in a HIF-1A-dependent manner in a hypoxic environment [54]. Overexpression of miR-210 suppressed the abundance of RAD52, which plays a role in homology-dependent repair. Similarly, overexpression of miR-373 decreased RAD23B, the nucleotide excision repair protein, and RAD52. Under hypoxic conditions, both RAD52 and RAD23B are downregulated. There is also evidence that miR-210 plays a role in mitochondrial dysfunction in some lung cancers. MiR-210 suppresses the expression of subunit D of succinate dehydrogenase complex (SDH), one of the subunits of the electron transport chain complexes I and II, and thereby activates HIF-1 [55]. In addition, miR-210 most likely decreases the expression of ISCU (iron-sulfur cluster scaffold homolog) and COX10 (cytochrome c oxidase assembly protein), two key factors of the mitochondria electron transport chain and the tricarboxylic acid cycle, during hypoxia [56]. In contrast to the role mir-210 plays in allowing cells to survive during hypoxia, it also represses genes expressed under normoxia that are no longer needed to adapt in a hypoxic environment [57]. Because of its numerous identified roles in regulating the hypoxic response in cancer, miR-210 may be a prognostic biomarker for several cancers including pancreatic [58], head and neck [59], and renal cancer [60].

One question that remains unanswered is whether hypoxia-inducible factors (HIF), the master regulators of the response to low oxygen, regulate the expression of the miRNAs. The HIF transcription factors contain an oxygen-regulated alpha subunit and a constitutively expressed beta subunit. Under normoxic conditions, the alpha subunit is degraded. Under hypoxic conditions, however, the HIF is stabilized and thus regulates the transcription of numerous genes including some involved with angiogenesis, glucose metabolism, and survival, all of which are likely to play a role in cancer development [61]. Suggestive evidence that HIF does regulate the transcription of hypoxia-inducible miRNAs under low oxygen conditions used chromatin immunoprecipitation assays to show that HIF binds to the promoters of regulated miRNAs [42]. It is unknown which factors function in regulating the miRNAs that are downregulated in hypoxia [46].

Another intriguing potential role for miRNAs in the microenvironment has recently been revealed. Solid tumors have an irregular vasculature that creates an environment that alternates between hypoxia and reoxygenation that most likely plays an important role in the tumor's survival. A subpopulation of cancer stem cells was identified under hypoxic and reoxygenating cycling conditions that are expected to function in relapse and metastasis by allowing new tumors to arise [62]. The expression of miR200c, miR205, and miR-215 is reduced under these alternating conditions in stem cells. MiR200c and miR205 were previously shown to be reduced in cancer stem cells [63]. MiR-215 suppresses epithelial-mesenchymal transition (EMT) by decreasing the expression of the mesenchymal transcription factor ZEB2 and increasing the E-cadherin level [64].

In most cases, the downregulation of particular miRNAs in regulating the hypoxic response has not yet been identified. An exception to this is found in hepatocellular carcinoma tissue where the functional consequence of reducing miR-29 expression sensitized cells to apoptosis that was triggered by hypoxia, and thus it is potentially useful as a cancer therapy [65].

## 5. The Need for Further Research in Understanding the Role of miRNAs in the Microenvironment

One of the key issues that need to be addressed in future studies is how miRNA biogenesis is regulated in the microenvironment of cancers. We are just beginning to identify the regulators of miRNA biogenesis (recently reviewed in [66–70]). RNA polymerase II, and in some cases RNA polymerase III, transcribes the primary transcripts (pri-microRNAs) [71, 72]. The pri-microRNAs also undergo capping, splicing, and polyadenylation and thus have many potential steps where regulation of expression might be controlled by endogenous and exogenous stimuli. It is less likely that the microRNA biogenesis machinery is responsible for the differential expression of miRNAs observed under hypoxic conditions since the miRNA processing proteins Ago2, Drosha, Exp5, Dicer, and DP103 expression is unaltered in hypoxic trophoblasts [45].

Another area where more research is needed is identification of the targets of the miRNAs. There are many web sites available that may be used to predict potential targets of the miRNAs. It is not unusual to identify hundreds of potential targets for a particular miRNA. In addition, comparison of the prediction between various web sites indicates that there is some, but not necessarily extensive, overlap between the predicted targets. Thus, it remains unclear which web sites are the most useful for predicting miRNA targets that will be experimentally validated. Perhaps a better approach with regard to studying the targets of the miRNAs is to identify mRNAs and/or proteins that are dysregulated under hypoxic conditions. Transcripts that are dysregulated under low oxygen and have predicted hypoxic regulated miRNA binding sites are excellent candidates for further testing.

Further investigation is also needed to determine if there are synergistic effects of miRNAs. In most studies in which the function of miRNAs is assayed, one miRNA at a time is either up- or downregulated. This approach will not allow the investigator to determine if the activity of each miRNA has any synergistic or inhibitory impact on the other miRNAs. Further studies are needed in which the expression of multiple miRNAs is manipulated so that the effects may be compared to the results obtained from manipulating the activity of individual miRNAs.

## 6. Conclusions

*In situ* carcinomas that remain localized and noninvasive at the time of diagnosis are associated with positive clinical outcomes and increased disease-free survival for patients. Unfortunately, the majority of tumors at the time of diagnosis are invasive malignant tumors with a portion of these having metastasized to secondary sites decreasing patient prognosis and survival. The ability of tumor cells to accomplish this migration and invasion is dependent on cross-talk with the surrounding microenvironment. An understanding of the mechanisms by which tumor cells communicate with the microenvironment to promote cancer growth and metastasis has the promise of providing innovative avenues of therapeutic intervention. Collectively, observations that miRNAs act as mediators of heterotypic signaling in the tumor stroma and ECM as well as influence responses to hypoxia provide researchers with a novel target for such therapies. The use of chemopreventative measures to control the expression of miRNAs in the microenvironment will be an important approach to the global control of cancer.

## Figures and Tables

**Table 1:** miRNA regulation in the tumor microenvironment.

miRNA	Up- or downregulated	Tumor microenvironment	Tumor entity	Reference
miR-16	Up	CAF	Bladder	[11]
miR17/20	Down	CAF	Breast	[30]
miR-21	Up	ECM, Hypoxia	Bile duct, breast, glia	[36–38, 42]
miR-29b	Up	CAF	Endometrial	[18]
miR-29c	Down	ECM	Nasophayngeal	[40]
mirR-31	Down	CAF	Endometrial	[18]
miR-143	Down	CAF	Bladder	[11]
miR-145	Down	CAF	Bladder	[11]
miR-146a	Up	CAF	Endometrial	[18]
miR-146b	Down	ECM	Glia	[39]
miR-320	Up	CAF	Bladder	[11]
miR-503	Up	CAF	Endometrial	[18]
